# Genome Sequences of 18 Salmonella enterica Serotype Hadar Strains Collected from Patients in the United States

**DOI:** 10.1128/mra.00522-22

**Published:** 2022-08-29

**Authors:** Hattie E. Webb, Justin Y. Kim, Kaitlin A. Tagg, Fernando de la Cruz, Arancha Peñil-Celis, Beth Tolar, Zachary Ellison, Colin Schwensohn, Joshua Brandenburg, Megin Nichols, Jason P. Folster

**Affiliations:** a ASRT, Inc., Suwanee, Georgia, USA; b Division of Foodborne, Waterborne, and Environmental Diseases, Centers for Disease Control and Prevention, Atlanta, Georgia, USA; c Instituto de Biomedicina y Biotecnología de Cantabria, Universidad de Cantabria, Santander, Spain; d Oak Ridge Institute for Science and Education, Oak Ridge, Tennessee, USA; University of Maryland School of Medicine

## Abstract

Despite being linked to a number of recent poultry-associated outbreaks in the United States, few reference genomes are available for Salmonella enterica serotype Hadar. Here, we address this need by reporting 18 Salmonella Hadar genomes from samples collected from patients in the United States between 2014 and 2020.

## ANNOUNCEMENT

Salmonella enterica serotype Hadar infections in humans in the United States increased in 2020 and 2021, compared with previous years, despite an overall decline in reported salmonellosis cases (1). Many infections occurred as part of recent outbreaks linked to either backyard poultry flocks (e.g., chickens and ducks) or consumption of ground turkey, but isolates linked to these different sources demonstrated a high degree of core genome relatedness (1, 2). Exploring the accessory genome may improve strain differentiation, as well as our understanding of the recent increase and evolution of this serotype. Here, we generated assemblies for 18 *S*. Hadar isolates collected from U.S. patients to serve as references for future investigations.

Briefly, isolates originated from clinical diagnostic laboratories or public health laboratories (PHLs) as part of the Centers for Disease Control and Prevention (CDC) national passive Salmonella surveillance (https://www.cdc.gov/nationalsurveillance/salmonella-surveillance.html); therefore, isolation methods varied by site (3). Isolates underwent short-read sequencing (https://www.cdc.gov/pulsenet/pathogens/wgs.html), and serotypes were confirmed using SeqSero2 v0.1 (4). Genomes were screened for resistance determinants and plasmids using the ResFinder database (downloaded 30 July 2020) (90% identity and a 50% cutoff value), the PointFinder scheme for Salmonella spp. (downloaded 30 August 2019) implemented in staramr v0.4.0 (5), a modified PlasmidFinder database (90% identity and 60% coverage) (https://cge.cbs.dtu.dk/services), and COPLA (6). Sequence types (STs) were determined using staramr v0.4.0 (with MLST software [https://github.com/tseemann/mlst] and PubMLST [7]). This report is a product of activities approved by the CDC internal review board (approval number 7172).

Isolates were selected for long-read sequencing based on diverse accessory genome content. Genomic DNA was extracted (Wizard genomic DNA purification kit [Promega, Madison, WI, USA], with a modification of the manufacturer’s protocol) from cultures that had been incubated on tryptic soy agar-sheep blood overnight at 37°C. Libraries were prepared using the rapid barcoding kit (SQK-RBK004; Oxford Nanopore Technologies [ONT], Oxford, UK) according to the manufacturer’s protocol and sequenced for 72 h on a GridION sequencing platform (R9.4.1 flow cells; ONT). Reads were base called using Guppy v4.2.2 and filtered for quality using MinKNOW (ONT). Hybrid assemblies were generated, polished, circularized, and rotated using Unicycler v0.4.8 (conservative option) (8); corresponding Illumina short reads that had been previously generated at the PHL (BioNumerics v7.6 [Applied Maths NV, Sint-Martens-Latem, Belgium] quality control metrics: quality score, ≥30; coverage, ≥30×) were accessed through NCBI (Table 1). Assemblies were quality controlled using QUAST v5.0.2 (9) and BLASTn v2.9.0 (10) and were annotated using the NCBI Prokaryotic Genome Annotation Pipeline (PGAP) v6.1 (11). Default parameters were used for all software unless otherwise specified.

**TABLE 1:** Summary information for 18 Salmonella enterica serotype Hadar (ST33) genomes from samples collected from patients in the United States

Strain	Collection yr	Accession no.				PTU (plasmid replicon)^*a*^	Antimicrobial resistance determinants	Short-read findings		Long-read findings				GC content (%)	Total size (bp)	Mean coverage (×)
		BioSample	Short-read SRA	Long-read SRA	GenBank			Mean read length (bp)	No. of reads	Contig *N*_50_ (bp)	Mean read length (bp)	No. of reads	No. of contigs			
2014AM-1331	2014	SAMN05596322	SRR4044556	SRR19768540	CP093126	—	*aph(3″)-Ib*, *aph(6)-Id*, *tet*(A)	295.9	1,617,299	4,702,738	4,302	391,542	3	52.26	4,741,847	170
					CP093127	PTU-N3	*—*									
					CP093128	PTU-E10	*—*									
2014AM-2067	2014	SAMN05596277	SRR4044454	SRR19768539	CP093122	—	*aph(3″)-Ib*, *aph(6)-Id*, *tet*(A)	286.7	1,838,942	4,763,043	5,225.5	165,516	4	52.22	4,777,204	192
					CP093123	PTU-E22	*—*									
					CP093124	PTU-E1 (ColE1)	*—*									
					CP093125	PTU-E19 (Col156)^*b*^	*—*									
2015AM-0414	2015	SAMN07268462	SRR5740069	SRR19768530	CP093120	—	*aph(3″)-Ib*, *aph(6)-Id*, *tet*(A)	278.3	1,430,061	4,801,674	5,580.5	174,520	2	52.25	4,805,578	148
					CP093121	PTU-E19 (Col156)	*—*									
2015AM-0511	2015	SAMN07415498	SRR5868150	SRR19768529	CP093140	—	*aph(3″)-Ib*, *aph(6)-Id*, *tet*(A)	274.7	1,566,516	4,778,352	5,993	223,197	7	52.21	4,805,332	162
					CP093141	PTU-E3 [Col440II,Col(pHAD28)]	*bla*_TEM-1C_									
					CP093142	PTU-E7 (Col156)	*—*									
					CP093143	PTU-E1 (ColE1)	*—*									
					CP093144	PTU-E58 (ColE1)	*—*									
					CP093145	PTU-E58	*—*									
					CP093146	PTU-E11 (ColpVC)	*—*									
2016AM-0673	2016	SAMN13512702	SRR10607636	SRR19768528	CP093116	—	*aph(3″)-Ib*, *aph(6)-Id*, *tet*(A)	277.9	1,363,160	4,703,663	5,196.8	324,412	4	52.29	4,712,319	143
					CP093117	PTU-E10	*—*									
					CP093118	PTU-NA	*—*									
					CP093119	PTU-E11	*—*									
2016K-0377	2016	SAMN05250424	SRR3667804	SRR19768533	CP093076	—	—	274.0	1,081,951	4,685,556	5,555.7	234,342	5	52.19	4,730,499	102
					CP093077	PTU-X1 (IncX1)	—									
					CP093078	PTU-E7 (Col156)	—									
					CP093079	PTU-E3 [Col(pHAD28)]	—									
					CP093080	PTU-E11 (ColpVC)	—									
2017AM-0493	2017	SAMN17129770	SRR13277812	SRR19768526	CP093109	—	—	298.6	866,278	4,819,027	5,104.1	104,476	3	52.18	4,829,291	90
					CP093110	PTU-E22	—									
					CP093111	PTU-E1 (ColE1)	—									
2021K-0017	2020	SAMN17478013	SRR13496954	SRR19768531	CP093072	—	*aph(3″)-Ib*, *aph(6)-Id*, *tet*(A)	272.7	662,153	4,711,128	4,785.5	459,695	1	52.27	4,711,128	67
PNUSAS002131	2016	SAMN04961841	SRR3499746	SRR19768527	CP093112	—	—	289.4	2,242,755	4,683,655	5,252.9	345,694	4	52.17	4,807,169	173
					CP093113	PTU-I1 (IncI1-Iγ)	—									
					CP093114	PTU-X1 (IncX1)	—									
					CP093115	PTU-E10 (Col8282)	—									
					—	(ColpVC)^*c*^	—									
					—	[Col(pHAD28)]^*c*^	—									
PNUSAS018090	2017	SAMN07427456	SRR6014222	SRR19768524	CP093096	—	—	294.5	977,909	4,685,429	5,517.2	241,397	7	52.17	4,764,800	88
					CP093097	PTU-X1 (IncX1)	—									
					CP093098	PTU-N3	—									
					CP093099	PTU-NA [Col(pHAD28)]	—									
					CP093100	PTU-E19 (Col156)	—									
					CP093101	PTU-E3	—									
					CP093102	PTU-E11 (ColpVC)	—									
PNUSAS021403	2017	SAMN07521433	SRR5951569	SRR19768525	CP093103	—	—	284.6	828,218	4,685,480	4,125.7	280,865	6	52.17	4,837,812	80
					CP093104	PTU-I1 (IncI1-Iγ)	*aadA2*, *ant(3″)-I*, *cmlA1*, *sul3*									
					CP093105	PTU-X1 (IncX1)	—									
					CP093106	PTU-E3	—									
					CP093107	PTU-E3 [Col(pHAD28)]	—									
					CP093108	PTU-E11 (ColpVC)	—									
PNUSAS037609	2018	SAMN08815166	SRR6916443	SRR19768523	CP093093	—	*aph(3″)-Ib*, *aph(6)-Id*, *tet*(A)	243.3	960,837	4,771,555	5,272.1	147,247	3	52.24	4,775,081	90
					CP093094	PTU-E58	*—*									
					CP093095	PTU-NA	*—*									
PNUSAS039582	2018	SAMN09011259	SRR7093175	SRR19768538	CP093088	—	*aph(3″)-Ib*, *aph(6)-Id*, *tet*(A)	276.7	2,708,767	4,801,678	6,900.7	314,036	5	52.27	4,908,883	252
					CP093089	PTU-NA (IncI1-Iγ)	*—*									
					CP093090	PTU-E1 [Col(pHAD28)]^*b*^	*—*									
					CP093091	PTU-E58 (ColE1)	*—*									
					CP093092	PTU-E10 (Col8282)	*—*									
					—	[Col(pHAD28)]^*c*^	*—*									
					—	[Col(pHAD28)]^*c*^	*—*									
PNUSAS067730	2019	SAMN11029788	SRR8643922	SRR19768537	CP093086	—	*aph(3″)-Ib*, *aph(6)-Id*, *tet*(A)	272.5	859,691	4,763,126	4,853.2	357,105	2	52.23	4,767,759	88
					CP093087	PTU-E11 (ColE1)	*—*									
PNUSAS074905	2019	SAMN11618493	SRR9040334	SRR19768532	CP093073	—	*aph(3″)-Ib*, *aph(6)-Id*, *tet*(A)	230.7	1,282,945	4,766,350	7,143.7	112,075	3	52.21	4,887,846	102
					CP093074	PTU-I1 (IncI1-Iγ)	*aac(3)-IId*, *ant(3″)-Ia*, *bla*_TEM-1B_, *tet*(A)									
					CP093075	PTU-E1 (ColE1)	*—*									
PNUSAS127695	2019	SAMN13795856	SRR10856733	SRR19768536	CP093135	—	*aph(3″)-Ib*, *aph(6)-Id*, *tet*(A)	263.0	612,925	4,801,677	5,894.7	376,137	5	52.24	4,819,091	62
					CP093136	PTU-E1 [Col(pHAD28)]	*—*									
					CP093137	PTU-E3 [Col(pHAD28)]	*—*									
					CP093138	PTU-E58 (ColE1)	*—*									
					CP093139	PTU-E1 (ColE1)	*—*									
PNUSAS147811	2020	SAMN15239347	SRR12017862	SRR19768535	CP093084	—	*aph(3″)-Ib*, *aph(6)-Id*, *tet*(A)	271.5	755,934	4,719,084	5,320.8	289,486	2	52.2	4,813,237	75
					CP093085	PTU-I1 (IncI1-Iγ)	*tet*(B)									
PNUSAS148096	2020	SAMN15249649	SRR12024307	SRR19768534	CP093081	—	*aph(3″)-Ib*, *aph(6)-Id*, *tet*(A)	129.2	737,381	4,708,042	4,754.8	293,015	3	52.23	4,802,435	41
					CP093082	PTU-I1 (IncI1-Iγ)	*—*									
					CP093083	PTU-NA	—									

a PTU, plasmid taxonomic unit; PTU-NA, plasmid taxonomic unit not assigned; —, no information.

b Plasmid assigned using updated version of COPLA.

c Plasmid replicon missing from long-read assembly but present in short reads.

All 18 *S*. Hadar strains were found to be ST33. Resistance determinants and plasmid types are summarized in Table 1. The most common resistance genes were *aph(3″)-Ib*, *aph(6)-Id*, and *tet*(A), which were always located on the chromosome (*n* = 13). When present, other resistance genes were associated with IncI1-Iγ or Col(pHAD28) plasmids. High levels of small plasmids with no known resistance genes were observed, some of which had not been previously characterized, as indicated by small, circular genetic elements not containing a known plasmid replicon. More generally, the hybrid assembly method employed here recovered small plasmids at a higher rate than did long-read-only assembly methods (data not shown). For two genomes, however, some small plasmids were not recovered despite the use of a hybrid assembly method (Table 1), a known artifact of the long-read sequencing process (12).

### Data availability.

The sequences discussed here have been deposited in GenBank and the SRA under the accession numbers listed in Table 1.
